# Modelling and Simulation Strategies for Fluid–Structure-Interactions of Highly Viscous Thermoplastic Melt and Single Fibres—A Numerical Study

**DOI:** 10.3390/ma15207241

**Published:** 2022-10-17

**Authors:** Benjamin Gröger, Jingjing Wang, Tim Bätzel, Andreas Hornig, Maik Gude

**Affiliations:** Institute of Lightweight Engineering and Polymer Technology, Technische Universität Dresden, Holbeinstraße 3, 01307 Dresden, Germany

**Keywords:** fluid-structure-interaction (FSI), finite element analysis (FEA), thermoplastic, composite, Arbitrary Lagrange–Eulerian (ALE), flow, fibre

## Abstract

A virtual test setup for investigating single fibres in a transverse shear flow based on a parallel-plate rheometer is presented. The investigations are carried out to verify a numerical representation of the fluid–structure interaction (FSI), where Arbitrary Lagrangian–Eulerian (ALE) and computational fluid dynamics (CFD) methods are used and evaluated. Both are suitable to simulate flexible solid structures in a transverse shear flow. Comparative investigations with different model setups and increasing complexity are presented. It is shown, that the CFD method with an interface-based coupling approach is not capable of handling small fibre diameters in comparison to large fluid domains due to mesh dependencies at the interface definitions. The ALE method is more suited for this task since fibres are embedded without any mesh restrictions. Element types beam, solid, and discrete are considered for fibre modelling. It is shown that the beam formulation for ALE and 3D solid elements for the CFD method are the preferred options.

## 1. Introduction

Continuous fibre-reinforced thermoplastic composites (TPC) are well established in industrial applications [1]. The main advantages of using thermoplastic matrix systems are an increased impact resistance [2], shorter cycle times in forming processes [3] and a wide range of available joining technologies for multi-material assemblies (cf. [4,5]). The high specific mechanical properties with adjustable direction-dependent stiffness behaviour especially enable a load-compliant design [6], which can be achieved by aligning the fibre direction of uni-directional single layers of TPC with the loading direction. However, handling and manufacturing of TPC typically result in process-induced fibre reorientation phenomena. Particularly joining or forming processes often involve large deformations which are accompanied by local fibre rearrangements such as wrinkles and discontinuities [7] or fibre reorientation and fibre fractures in the joining area [8,9,10,11,12]. Process-induced fibre reorientations are well investigated for short fibre applications, where injection moulding is used and a method for manipulation under magnetic fields is already available [13,14]. Discontinuities and reorientation of fibres resulting from moulding, forming and joining processes are the main source of uncertainties concerning the load-bearing capabilities of structural TPC components. Therefore, the prediction of such effects will allow for a more effective design process. Additionally, a more precise determination of process parameters is enabled, regarding, e.g., tool velocity and design, applied pressure, and process temperature aiming improved structural stiffness and strength while eliminating trial and error procedures. Furthermore, process simulations for the assessment of mixing phenomena on micro scale are carried out [15]. Within the simulations the influence of the shape mixing structure to the improvement of the mixing index is shown.

For the numerical modelling of complex forming processes needs to consider interactions between tool, fibres, and the molten thermoplastic matrix. The tool motion induced displacements of fibres and molten matrix. Furthermore, the interaction of viscous matrix and flexible fibres is driven by squeeze flow and percolation phenomena [16]. A prediction of the fibre behaviour under these flow conditions is a crucial aspect for complex process simulations to identify local regions of low fibre volume contents and diverging fibre orientations. Therefore, suitable numerical modelling strategies to describe the fluid-structure-interaction (FSI) have to be identified investigated. In the presented work the FSI between a flexible glass fibre (GF) (E= 73 GPa) and a highly viscous thermoplastic polypropylene (PP) melt (η≈ 200 Pa · s) is considered. Due to the high viscous melt and the flexible fibres the term of fibre-matrix-interaction (FMI) is introduced.

Available literature involving experimental investigations is mostly focused on analytical models and phenomenological descriptions for short and long fibres. For this purpose, a Couette flow is frequently used to investigate the fibre behaviour in shear flows [17,18]. The fibre length dependent deformation behaviour is mainly affected by bending and reorientation as presented in [19]. It was shown, that for short fibres a springy rotation of the whole fibre occurs. Whereas, with increasing fibre length, bending phenomena of the fibre ending in flow direction occur and propagate along the fibre, leading to, e.g., snake rotations. When the fibre exceeds a critical length a helix rotation can be observed [19].

Numerical investigations of flexible fibres in shear flows mainly focus short or long fibres. A distinction has to be made between single fibre [20,21,22] and multiple fibre modelling setups [18,23]. Established methods for fibre modelling are beam elements [21,23] or interlinked rigid spheres allowing elongation, bending, and twisting by a bear-spring chain model [20] or ball socket connection [24]. Instead of rigid spheres cylindrical segments can also be used [22,25,26,27]. There, linking is realised by ball and socket joints [22,25] or a viscoelastic material model [27]. For FSI one-way coupling between flexible structures and the shear flow is implemented by the immersed boundary method. In [18], flexible joints were used considering fibre breakage by bending. The input parameter of the critical bending radius was determined experimentally. A drawback of this modelling approach is that deformation due to tension, compression or shear can not be considered.

Furthermore, for more technological investigations, such as wind turbine blades, numerical studies are carried out in [28] to asses computational methods.

Flexible biological structures in flows of water are presented in [29]. The flow is directed transversely to the structures leading to bending superimposed by natural buoyancy. An analytical model relates the deformation behaviour with flow velocity, stiffness, and drag of the structure in a laminar flow.

Experimental and numerical setups typically use either Couette or laminar flows. For investigations of FMI in a Couette flow and fibres with a length above 20 mm the three dimensional (3D) reorientation has to be considered [19]. Test setups for fibres in a laminar flow require a surrogate fluid for simplifying the test setups (e.g., heater, lower temperatures), which leads to a lack of information caused by the difference in the shear rate induced behaviour of the surrogate fluid and the thermoplastic matrix. Furthermore, forming and joining processes often imply flow processes perpendicular to fibre orientation leading to bending in the joining zone.

To address the above mentioned limitations, a test setup based on a parallel-plate rheometer is proposed. In this study, the corresponding virtual testing environment is presented, which enables a detailed investigation of FMI of fibres with a length of 25 mm in a molten PP under defined transverse shear flow. The fibre is positioned in the middle of the shear gap and aligned radial to the cylindrical specimen. Due to the shear rate $\dot{\gamma }$ of 0 s^−1^ in the centre of rotation and a velocity *v* with ω·r at the boundary areas, the fibre is rotated and can be bent at the fibre endings. A modelling strategy is developed to investigate numerical methods for the description of the FMI. This enables more complex simulations of forming processes taking FMI into account. The chosen numerical approaches are based on Arbitrary Lagrangian Eulerian (ALE) and Computational Fluid Dynamics (CFD) methods using LS-DYNA. The preferred approach should enable a simulation of single as well as multi-fibre systems.

## 2. Materials and Methods

### 2.1. Materials Specification

**Figure 1:**
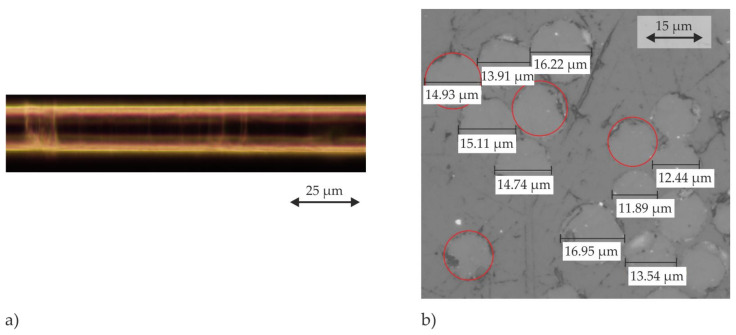
Micrographic characterisation of (**a**) a single fibre surface and (**b**) cross-sections of fibres to measure the fibre diameters.

The investigations are performed using glass fibre from Saertex^®^ (Nordrhein-Westfalen, Germany) with the engineering constants given in Table 1. For a qualitative evaluation of a single fibre surface and to determine the fibre diameter, a micrographic characterisation has been performed (Figure 1a,b). The fibre shows homogeneous reflections with small circumferential imperfections, indicating a sufficiently smooth surface. Therefore, effects of surface roughness on the FMI are neglected. A circular fibre cross-section with a diameter based on the measured mean value is assumed.

**Figure 2:**
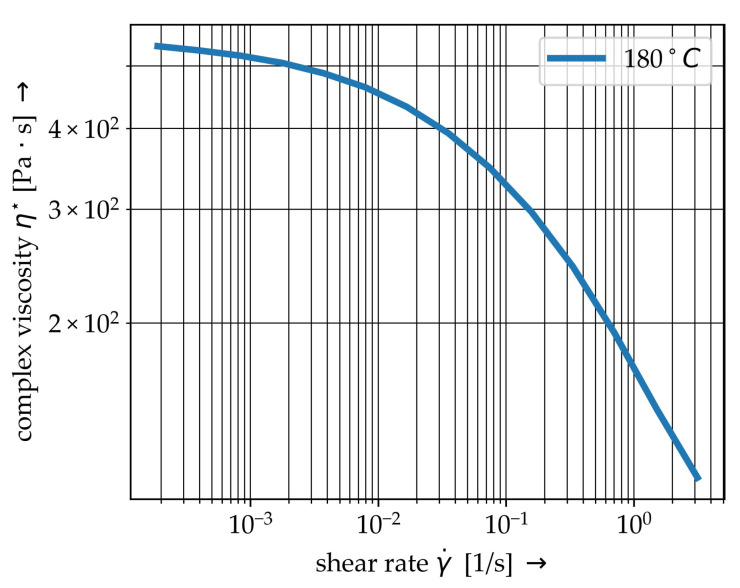
Shear rate dependent complex viscosity $\eta^{*}$ of the used PP BJ100HP at 180${}^{\circ }$.

The investigated PP thermoplastic matrix material is a BJ-100HP from Borealis^®^ (Austria, Vienna). Shear rate dependent complex viscosity $\eta^{*}$ are determined using a Anton-Paar MCR-502S oscillating parallel-plate rheometer setup. Results with respect to the investigated temperature level are given in Figure 2. The performed oscillation measurement can lead to a detachment of the molten matrix and rheometer plate, resulting in a phase shift caused by the material’s inertia and the higher shear rates [30]. It can be assumed, that the shear deformation and the oscillating measurement for higher shear rates are not capable when the shear rate is greater. Hence, the data points for $\dot{\gamma }>$ 3.14 s^−1^ are neglected.

### 2.2. Virtual Test Setup

**Figure 3:**
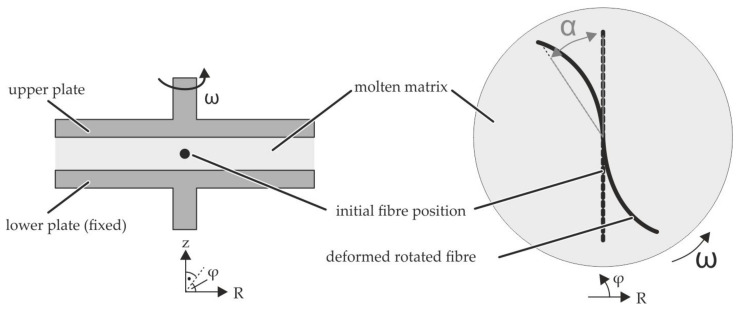
Principle of the parallel-plate rheometer test setup for single fibres in transverse shear flows.

**Figure 4:**
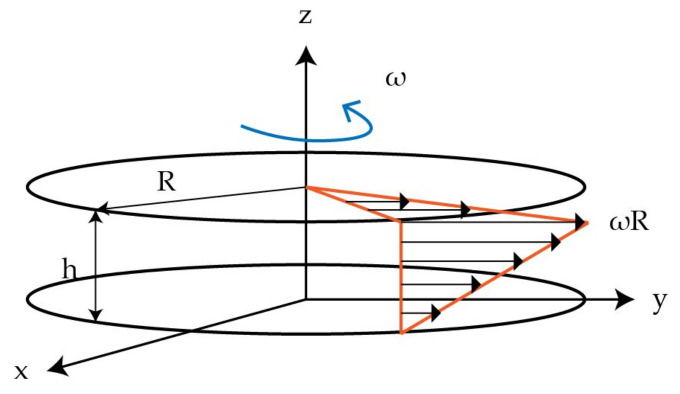
Velocity distribution of a parallel-plate rheometer (according to [31]).

To investigate the single fibre behaviour in a perpendicular the shear flow a parallel-plate rheometer was reproduced in a virtual modelling environment. The setup and the specimen positioning is shown schematically in Figure 3. The single fibre in the middle of the specimen is aligned in radial direction and has the specimen diameter (⌀D= 25 mm). A shear flow in circumferential direction is induced by rotation of the upper plate, while the angular velocity ω of the upper plate is kept constant, tangential/circumferential velocities increase with increasing radial distance to the centre point. Starting from zero velocity at the lower fixed plate, the velocity increases over the height toward the upper plate. These two superimposed profiles are illustrated in Figure 4. Due to the 3D velocity profile of the shear flow between both plates the whole fibre is rotated and additionally bent at the fibre endings (Figure 3 right side).

### 2.3. Numerical Methods

Modelling of flexible fibres in shear flows of molten thermoplastic matrix requires a coupling of a flexible solid structure and a highly viscous fluid to address information transfer between the fluid (pressure *p* and velocity *v*) and the solid domain (stress σ and deformation *u*) [32]. Several approaches can be chosen depending on coupling direction and coupling strength. The first method is an interface-based approach, which is used for the ICFD modelling technique in LS-Dyna. The mesh for the solid structure and the fluid domain mesh are spatially separated. Information transfer is enabled by an interface shell between the meshes. This method has been increasingly applied in the FSI field in recent years [32,33,34]. A second approach is called the ALE approach in LS-Dyna. The FSI is taken into account by the embedding of a solid structure domain mesh into the fluid domain mesh and constraining the two materials [35,36,37]. In the presented paper both modelling strategies are studied.

#### 2.3.1. ALE Method

**Figure 5:**
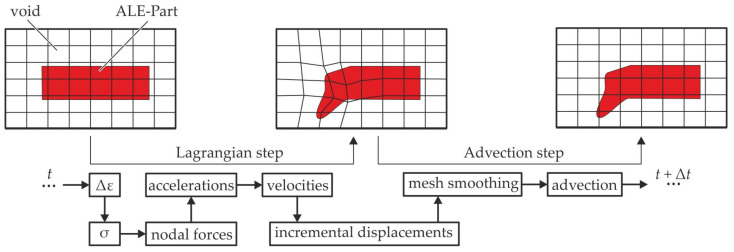
Mesh motion and calculation schedule of the explicit ALE approach for one time step Δt.

The ALE approach is based on explicit time-integration and a mesh domain, which allows elements and material to move independently. A calculation scheme consisting of two steps for each time increment is followed (Figure 5). After initially filling the elements partly with material, internal or external forces are applied in the first step, usually referred to as Lagrangian step. Based on the resulting strains and incremental displacements, the elements and nodes move accordingly. To avoid mesh distortion an advection step is added. In this mesh smoothing step the nodes (and elements) are moved into the initial spatial coordinates. The material flow based on this smoothing is calculated and the subsequent time increment is initiated. The advection step is often leading to simulation termination and numerical instabilities in terms of negative volumes. Therefore, a modified Van Leer advection logic is used (cf. [38,39]). Furthermore, an hourglass control (TYPE 6) is added. Two different modelling approaches can be used for the ALE method: void based (cf. [40]) and ALE Multi Material Group based (cf. [37]). In the present work the void approach is used. The final setting parameters for the final model are reported in Appendix A.

FMI is realised by penalty-based coupling method for solid structures in fluid domains called CONSTRAINT_LAGRANGIAN_IN_SOLID (CLIS) for solid element meshes or penalty CONSTRAINT_BEAM_IN_SOLID (CBIS) for idealised beam chains in LS-Dyna. Therefore the solid mesh is embedded into the fluid domain mesh. This coupling technique neither require consistent meshes nor boundary layers around the solid structures. Within these constrained-based coupling methods the momentum balance will be preserved so that the nodal velocities of the different element types (solid and fluid) follow each other. This can lead to a lack of energy. For the penalty-based method, forces are used to describe the coupling similar to standard contact definitions. Depending on the distance between solid and fluid a pressure is generated which leads to a coupling force.

#### 2.3.2. Incompressible Fluid Solver

The ICFD-solver for incompressible fluids is an implicit CFD-tool for calculating flow with the Navier–Stokes-Equation (NSE). Within a given fluid domain the meshing is automatised by the solver and updated between time increments. For separating CFD mesh and solid structure mesh domains, a third interface-domain (shell mesh) is generated. Due to the high viscosity and the expected viscous force an additional boundary layer at the interface shell is implemented in the presented models. Therefore, there is not only pressure force but also viscous force at the boundary layer taken into account [41]. At this interface pressure *p* and velocity *v* is transferred to the solid structure domain, while establishing an equilibrium with these external forces on the solid structures boundary, a deformation *u* is created. This deformation *u* is back-fed to the interface-domain [32] and the CFD mesh is updated accordingly. The calculation of the following time step starts. Major advantages of the ICFD-solver is the accurate handling of the fluid with the NSE and a variety of available material models to represent the shear rate dependent fluid behaviour. Furthermore, either one way or two way coupling can be chosen.

## 3. Numerical Setup

### 3.1. Material Modelling

For numerical simulations of FSI a flexible solidified fibre in a high viscous molten matrix has to be modelled. Therefore, elastic and viscosity based material models are used.

#### 3.1.1. Molten Matrix

Shear thinning behaviour above melting temperature has to be considered for PP (cf. Figure 2). Therefore, the MAT_ALE_02-ALE_VISCOUS model is used in the ALE method. This material model allows a definition of the shear-rate dependent dynamic viscosity based on a user-defined table. The viscosity is used to determine the deviatoric stresses [42]

(1) $$\sigma_{ij}^{\prime }=2\;\cdot \;\eta {\dot{\epsilon }}_{ij}^{\prime }\;$$

with dynamic viscosity η and deviatoric strain rate tensor $\epsilon_{ij}^{\prime }$.

The resultant stress and strain of molten matrix material is related by [43]:

(2) $$\sigma_{ij}=-p\;\cdot \;\delta_{ij}+2\;\cdot \;\eta {\dot{\epsilon }}_{ij}^{\prime }\;,$$

considering deviatoric and hydrostatic stress components with the identity matrix $\delta_{ij}$. In addition to the viscous fluid definition an Equation of state(EOS) has to be defined for the ALE approach in order to calculate hydrostatic components of the stress tensor. The common Grüneisen-EOS (*EOS_GRUNEISEN) with a simplified description is chosen:

(3) $$p=\frac{\varrho_{0}\;\cdot \;C^{2}\mu_{EOS}\left[1+\left(1-\frac{\gamma_{EOS}}{2}\right)\right]\mu_{EOS}}{{\left[1-\left(S_{1}-1\right)\mu_{EOS}\right]}^{2}}+\gamma_{EOS}\;\cdot \;E_{i}\;,$$

with $\mu_{EOS}$ as the volumetric strain, $\gamma_{EOS}$ as the Grüneisen coefficient, $E_{i}$ as the internal energy and $S_{1}$ as a specific material parameter. Calculated parameters are listed in Table 2. For the used model ρ the density, a pressure cut-off PC to allow cavitate below PC and MULO as the user-defined shear rate-dynamic viscosity curve [42] based on Figure 2 is given [31].

**Figure 6:**
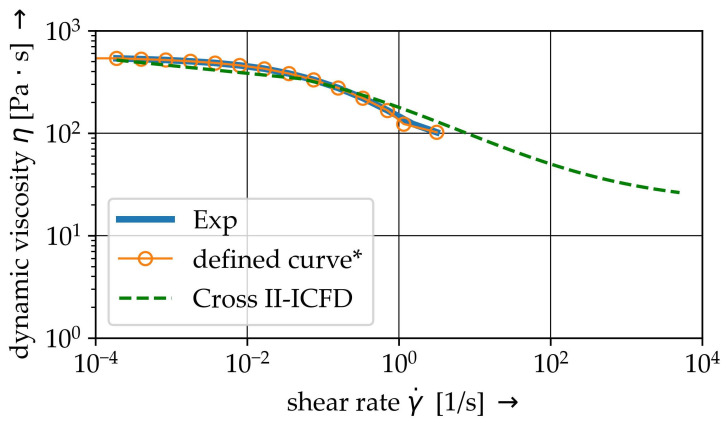
Shear-rate viscosity curves of the experiments, the user defined curve* (ALE) and the CROSS II- approach for the ICFD solver. (*infinity viscosity $\eta_{\infty }$ of 20.6 Pa · s at $\dot{\gamma }=4\times 10^{35}s^{-1}$ for numerical stability is not printed).

Due to the lack of information on viscosity parameters for shear rates above 3.14 s^−1^, generic value of 20.6 Pa · s for $\eta_{\infty }$ according to the curve fit of ICFD approach is defined. Thereby numerical instabilities caused by an overestimation of the viscosity at high shear rates are avoided. The defined curve is illustrated by Figure 6.

For the material modelling within the ICFD-Solver different models for non-Newtonian behaviour are available. Due to relatively low shear rates $\dot{\gamma }$ in comparison to some industrial technologies, e.g., injection moulding, the CROSS model is used:

(4) $$\eta =\eta_{\infty }+\frac{\left(\eta_{0}-\eta_{\infty }\right)}{1+{\left(\lambda \dot{\gamma }\right)}^{n}}\;,$$

with the given Newtonian viscosity $\eta_{0}$, the given infinite shear viscosity $\eta_{\infty }$, the time constant λ and the power law index *n* are defined. Those parameters were determined though curve fitting, see Table 3. The resultant shear rate—viscosity—curve is plotted in Figure 6.

#### 3.1.2. Glass Fibre

The numerical discretisation of relative brittle GF is realized in different three ways: (1) beam chain, (2) Discrete Element Method (DEM) and (3) solid elements. The fibre is assumed to be linear elastic. Due to the shear flow and small resultant viscous force, a fibre failure is not considered. Therefore, an ideal elastic behaviour (*MAT_001-ELASTIC) is assumed. The geometric parameters and Young’s modulus are determined through micrographs and external data (cf. Table 1).

For DEM modelling the keyword *DEFINE_DE_BOND is added to define elastic interaction between discrete element chains. These interactions of bonded discrete element chains represent the fibre stiffness. Therefore, the stiffness in normal direction PBN, is set equal to the Young’s modulus *E* and in transverse direction PBS is set:

(5) $$PBS=\frac{G}{E}=\frac{1}{2(1+\nu )}\;,$$

where *G* is the shear modulus, *E* is the Young’s modulus and ν is the Poisson’s ratio. Further, an interaction radius MAXGAP is defined. The parameter has to be greater than the distance between two elements to generate a coupling.

#### 3.1.3. Void

Following the ALE approach, a spatial mesh domain around the molten area needs to be defined. Depending on the modelling method (cf. [40]) a void model has to be used.

### 3.2. Modeling Strategy

For the development of an appropriate modelling strategy a detailed analysis and evaluation of the different approaches is conducted using simplified numerical setups. In Table 4, the strategy with the different models and the objectives is presented. With the scope of more complex use cases with advanced textile architectures, fibres, fibre bundles and yarns are considered. For this purpose, a collision model based on a compaction use case is developed (1). The setup enables also a investigation of the contact description between elastic solid structure and the modelling of the embedded structures within the ALE fluid domain. The second model enables the analysis of the flow behaviour surrounding an elastic structure (2). Especially the influence of the structure modelling on the velocity profiles investigated. The complexity of the flow profile and the boundary conditions is increased in (3). Due to the two dimensional (2D) shear flow the modelling strategy and applied boundary conditions are investigated. The fully 3D rheological FSI model (4) based on the findings of the previous models. With final model the influence of changed boundary conditions (velocity) and the resultant deformation of the structure are analysed. It should be noted that the three simplified models (1), (2), and (3) are set up to study the specific problems in the FSI model, e.g., the contact between fibres and the coupling between solid structure and fluid, so the numerical parameters such as model size and boundary conditions are designed according to the needs of problems. Therefore, for every model the mesh information in terms of characteristic element length $l_{e}$ and number of elements for the solid and the fluid domain are given.

#### 3.2.1. Collision Model

**Figure 7:**
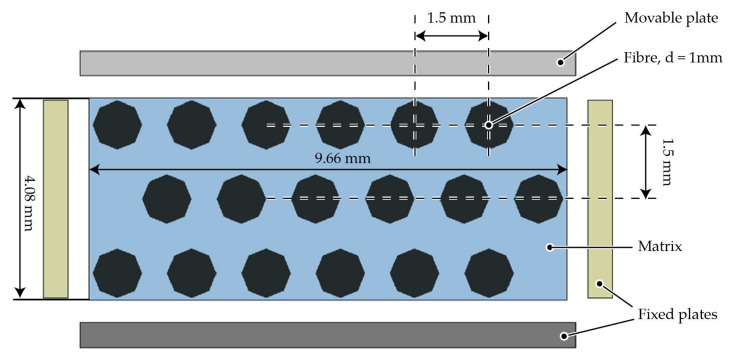
Geometric model setup for the collision test.

This setup aims at investigating a modelling strategy capable of handling more than a single fibre must be handled in the simulations. Therefore, the fibre–fibre interaction via contact modelling has to be considered. Since there is a large number of contacts between the fibres, the term collision is more appropriate. A simplified collision model is adapted from forming processes simulations (cf. [40]). It is based on a squeeze flow process (Figure 7) with a depth of 10 mm. An upper plate moves downwards to a lower fixed plate. Between the plates three rows of fibres are embedded in a fluid domain with a constant viscosity. Due to the non existing boundary of the fibres, two fixed plates are positioned on each side. Additionally, a gap between the three fixed plates (side and lower plates) allows for a transverse outflow of the fluid. The outflow avoids hydro static stress states in the cavity and provides fibre–fibre-contact when the fluid is squeezed out. For the investigation of collisions, the three different discretisation methods as described in Section 3.1.2 with different characteristic elements lengths are used. The specific parameters are summarized in Table 5. FMI is realised by CBIS in the beam model and CLIS in the solid and DEM model.

#### 3.2.2. Laminar Channel Flow

**Figure 8:**
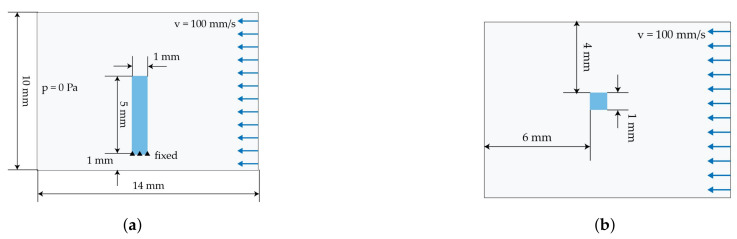
Simplified model of a flow channel. (**a**) Side view. (**b**) Top view.

For the next stage of preliminary investigations, a numerical model of a channel with a laminar flow is used to compare the flow distribution around the elastic structures. ALE results are compared to the results of ICFD and the accurate solution of NSE. Furthermore, different mesh types of the flexible fibres are investigated. The deformation of the embedded solid structure is assumed to be negligible in this setup. The solid structure is positioned in the flow channel, where the lower end is fixed. The saturated flow of the fluid is defined with an inflow velocity *v* of 100 mm · s^−1^, a constant viscosity of 200 Pa · s and an outflow of 0 Pa. The dimension of the structure and flow velocity is chosen in accordance to a yarn structure of continuous fibre-reinforced composites and a low tool velocity in joining processes [8]. The model with a characteristic element length $l_{e}$ of 1 mm is illustrated in Figure 8. Meshing details are given in Table 6.

The FSI forces for each solid structure node is tracked in case of the ALE method, whereas the ICFD model enables the distinction of pressure induced drag forces and viscous drag forces using *ICFD_DATABASE_DRAG for the whole interface. For a better comparison of the two methods, only nodal forces at the tip of the flexible fibre are considered.

#### 3.2.3. Shear Flow Model

**Figure 9:**
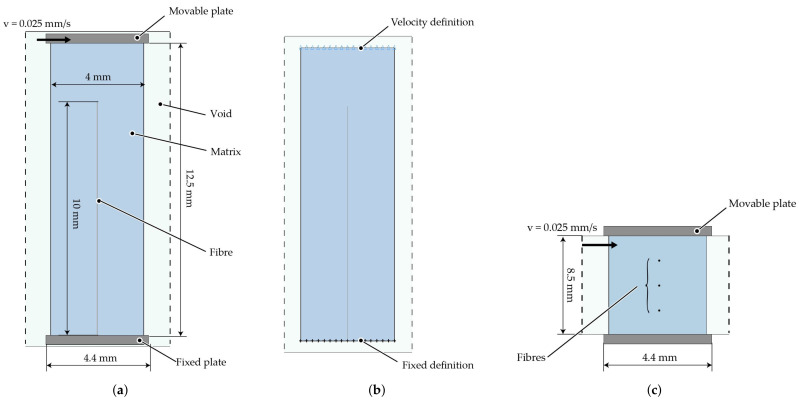
Simplified model of a 2D shear flow in vertical and horizontal direction with an embedded beam structure. (**a**) Vertical ALE model. (**b**) Vertical ALE model with an initial flow definition. (**c**) Horizontal ALE model.

The shear flow model is based on the transformation of the 3D flow in the rheometer shear gap (cf. Figure 4) into a 2D flow. For this purpose, a vertical (Figure 8) and a horizontal model (Figure 9c) are generated. In the vertical model, the fibre is vertically placed and the radial velocity profile of the rheometer is used. The velocity is based on the tangential velocity Equation (6)

(6) v=ω·r,

with distance *r* to the center of rotation and the circular frequency ω. This low velocity *v* equivalent to ω= $2\times 10^{-3}$ 1/s and therefore a boundary for experimental test setups. The shear flow leads to a bending of the fibre at the top, whereas the bottom is fixed in space. Furthermore, studies using different types of velocity definitions are carried out. In the first one, the cavity plates are positioned at both the upper and the lower end (Figure 9a). The upper plate is moved and initiates a horizontal flow with a FSI induced velocity boundary condition. The other type uses a direct definition of the velocity at the ALE elements at the top and bottom leading also to a horizontal flow (Figure 9b). The advantage of the model is the reduction of the interaction between the plates. Both, the FSI induced and the direct defined velocities have the some order of magnitude. The two resultant flow profiles and the deflection of the fibre are compared.

In the horizontal model three fibres are positioned horizontal to the flow in different heights (Figure 9c). Due to the 2D velocity profile from the bottom to the upper plate, the resultant velocity at a given height is constant. Therefore, at each fibre in a specific height the velocity along the fibre is constant and as well as the coupling force between fluid and fibre elements. Thus, leading to a shifting of the whole fibre depending on the resultant velocity. In conclusion, no bending effects should be observed.

The investigations are carried out with a constant viscosity η of 200 Pa · s. This allows a direct comparison without any shear rate effects. Furthermore, the maximum velocity *v* of 0.025 mm · s^−1^ at the top of the cavity is kept constant for each model. Table 7 summarizes the meshing details of the model.

### 3.3. Rheological FSI Model

**Figure 10:**
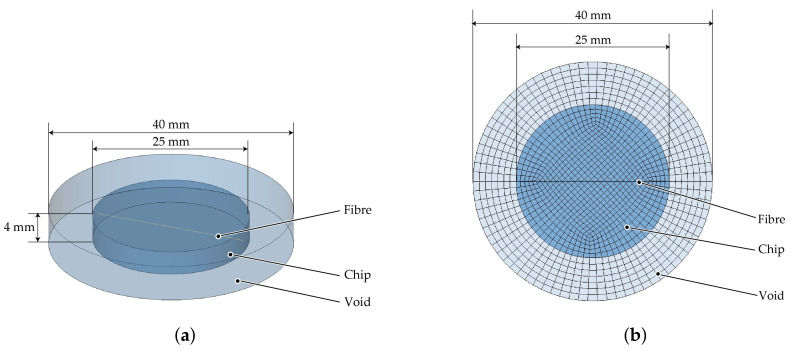
Numerical setup for the rheologicalFSI model with the ALE method. (**a**) Geometric structure of ALE model. (**b**) Mesh division of ALE model.

The rheological FSI model is the exact representation of the virtual setup (Figure 10). With respect to the specific modelling requirements, the ALE model has an Eulerian domain wider than the spacial dimension of the specimen. Hence, surrounding ALE elements of the fluid domain are defined as void (Figure 10a). The fibres have a diameter of 14 μm and are aligned in radial direction from one end to another (cf. Table 1) and are represented by beam elements. The mesh division of the model is shown in Figure 10b and mesh parameters are given in Table 8. The shear flow is generated by velocity boundary conditions (cf. Equation (6)) at the top elements of the fluid domain. The shear rate depended fluid behaviour is represented by the viscosity curve at 180 °C (cf. Figure 6). The ALE elements of the lower plane of the fluid domain are defined with a zero velocity condition. The angular frequency and the resulting velocity are in the same order of magnitude as when measuring the viscosity curves.

## 4. Results

### 4.1. Collision Model

**Figure 11:**
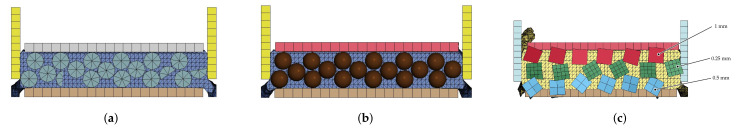
Contact behaviour of fibres with different elements types after compression with the ALE approach: (**a**) Beam elements; (**b**) discrete element chain; (**c**) 3D solid elements with varying element length. (**a**) Fibre arrangement after compression, beam element. (**b**) Fibre arrangement after compression, discrete element. (**c**) Fibre arrangement after compression, 3D solid element.

Beam and DEM modelling show similar results in terms of fibre arrangement after compaction in the collision model setup, see Figure 11a,b. As indicated by the absence of penetrations, both modelling approaches provide a sufficient representation of the interaction of polymer melt and fibre, as well the for fiber–fiber-interactions.

To ensure a sufficient element quality, fibres are approximated by a quadratic cross-section in the solid element model. Three different mesh densities were investigated: 1.0 mm, 0.25 mm, 0.5 mm (top to bottom in Figure 11c). Figure 11c indicates the last recoverable state before termination due to severe element distortions. The two coarse configurations (red and blue) featured significant hourglass modes, while even the finest mesh (green) exhibited high element distortions during contact with adjacent fibres. Moreover, penetrations of interacting elements of different fibres were observed. Thus, no stable solution of the contact interaction could be achieved. Hence, the impact of mesh densities regarding contact interactions needs to be investigated in more detail.

**Figure 12:**
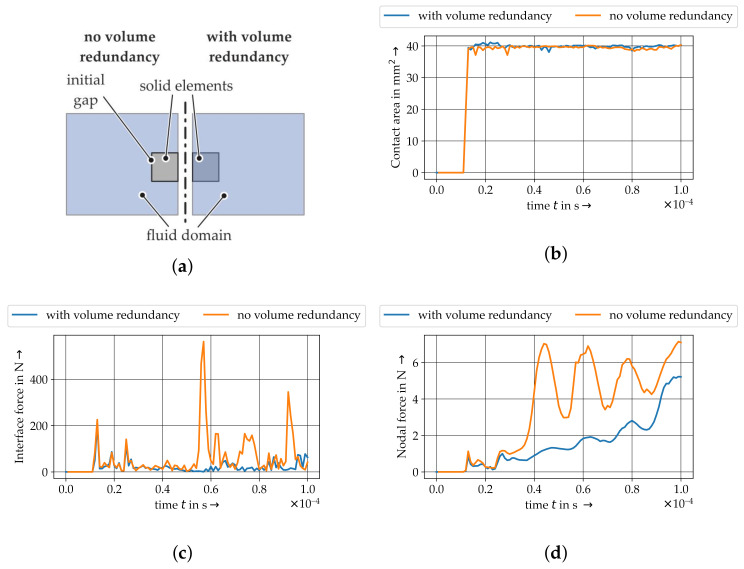
Investigations of the volume redundancy in ALE approach with a single structure in the collision model setup. (**a**) Principle of volume redundancy. (**b**) Contact area. (**c**) Interface forces. (**d**) Nodal forces.

Volume redundancy aspects are investigated with a reduced single fibre model (Figure 12a). Therefore, a single fibre within a 3D solid mesh (element length of 0.25 mm) is positioned between the plates. Only this element type allows an elimination of the volume redundancy due to the spacial dimension of the 3D element. Furthermore, the contact between element nodes and the fluid is necessary for the coupling algorithm. Therefore, a volume redundancy cannot be avoided for one dimensional element types. Two modelling techniques are carried out. Firstly, due to the embedding of a solid domain into the fluid domain, the volume of solid and fluid are overlapping resulting in a redundancy of volume and compaction. Secondly, a more intricate approach in terms of model preparation eliminates the overlapping volumes (no volume redundancy). The resultant contact area is shown in Figure 12b. The calculated contact areas are similar. Figure 12c shows the interaction force for the whole structure of both approaches. Both exhibit comparable interface forces but the model with eliminated volume (no volume redundancy) shows more variation at the interface, whereas the embedded model is less noisy (Figure 12c). The variation of nodal force at one point of the solid structure is plotted in Figure 12d. It is evident, that the eliminated volume model has a higher variation of contact force during compaction. The other model also shows slight variations but no overshooting. In conclusion, the volume redundancy leads to less dynamic effects at the interaction and may stabilise the numerical simulation. Therefore, the volume redundancy is used in the further models.

### 4.2. Laminar Flow Channel

**Figure 13:**
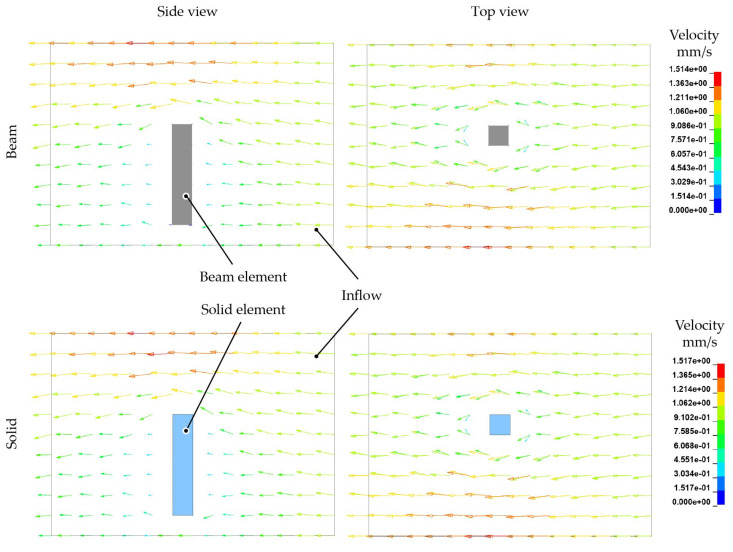
Similar flow behaviour around structures modelled with beam and solid elements (ALE method).

First, the flow behaviour of the melt is considered in ALE models with beam and solid model fibres, respectively. The discrete elements show no influence to the velocity field. Therefore, the modelling approach is not investigated in further work. The distinctive flow around the single fibre is illustrated by the vector plot in Figure 13. Flow fields and orientations are also similar for both models. It is concluded that beam and solid model interaction algorithms are able to describe the FSI likewise within the ALE framework.

**Figure 14:**
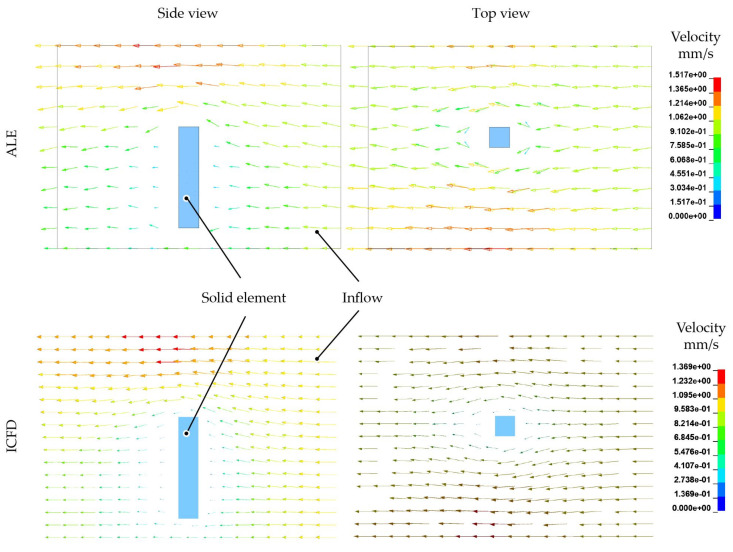
Flow behaviour around solid structures modelled with ALE and ICFD method.

The laminar flow channel model is used to draw a comparison between ALE and ICFD methods in the next step. Both models contain a single 3D solid element fibre. In general, the resulting velocity distributions with high values at the peripheral area and lower values close to the fibres are comparable. The ICFD model exhibits a maximum flow velocity that is in the same order of magnitude compared to the ALE model. It is important to note that the ICFD model contains an automatic mesh adaptation, which leads to the refined vector arrow distribution in Figure 14.

**Figure 15:**
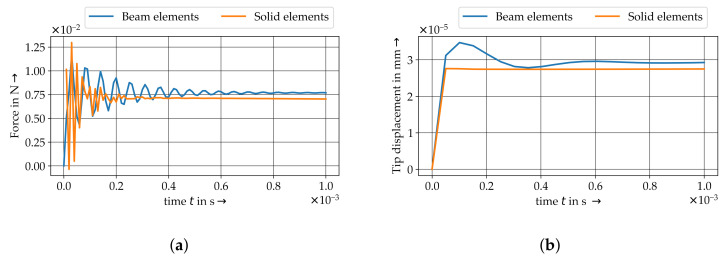
Resultant nodal force at the tip and tip deflection in flow direction of the flexible structure. (**a**) Resultant nodal force. (**b**) Tip displacement.

Despite the comparison of the flow and velocity profile, the resultant tip deflections and nodal forces at the top is shown in Figure 15. In accordance to the collision model, initial dynamic effects induced by the solid structure is reflected by the nodal force response. The resultant tip displacement decays immediately and stays constant. For the beam modelling technique a transient phenomena can be observed for force and tip displacement.

### 4.3. Shear Flow Model

**Figure 16:**
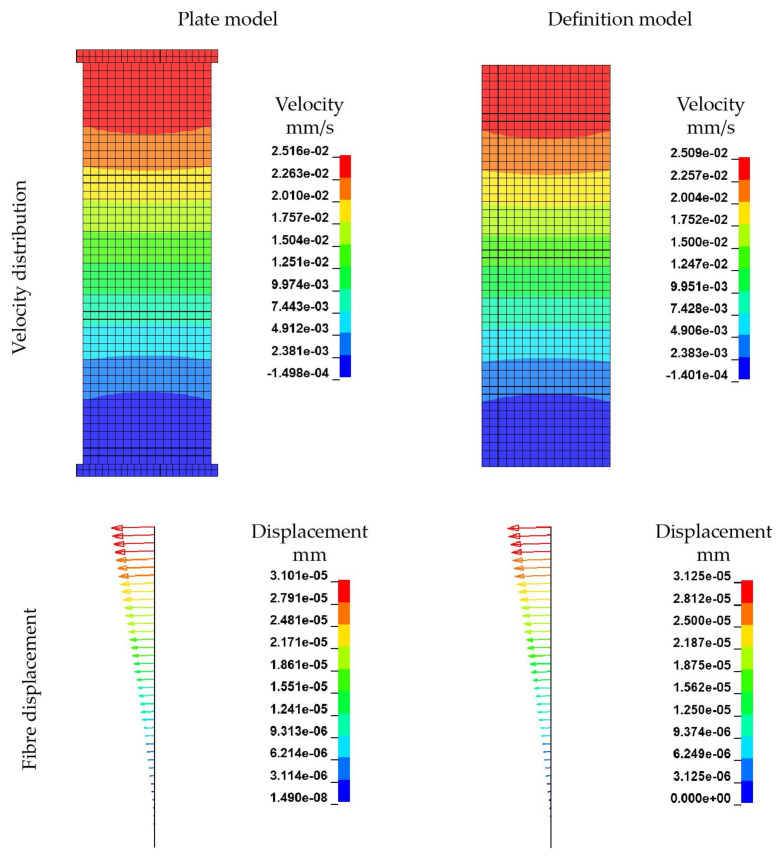
Influence of boundary condition for the velocity definition by plate (FSI) and direct definition.

**Figure 17:**
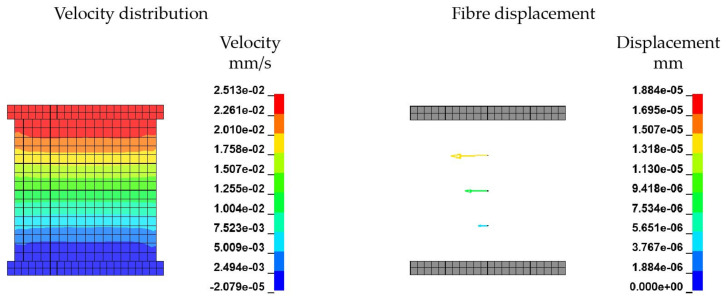
Results of vertical shear model.

The shear flow model results reveals a layered velocity distribution in thickness direction, see Figure 16 and Figure 17. As expected, the region of maximum velocity is located at the top edge of the melt volume where the moving plate or the velocity boundary condition is acting. Near the bottom edge the melt is close to static. This velocity field corresponds to the expected radial velocity profile of a parallel-plate-rheometer test. Both the plate model and the direct boundary condition model generate a coherent velocity field with negligible differences (Figure 16). Resultant translations of embedded fibres are also coherent (difference of maximum displacement < 1%). Thus, it is evident, that plates introducing the flow velocity can be substituted by a direct velocity boundary condition acting upon outer edges of the melt volume. Hence, the computational effort can be reduced and the modelling setup for more complex problems can be simplified.

Figure 17 illustrates the velocity field (left) and fibre displacement (right) of the vertical shear model and represents the plate-to-plate velocity distribution in typical shear rheometer tests. Three single fibres positioned at different heights between the plates are subject to displacements of varying magnitude depending on the plate-to-plate velocity gradient of the polymer melt. It can be seen, that the fibres are just shifted according to the increasing velocity and coupling forces with increasing height. The ALE method shows a high accuracy and sensitivity of the given boundary conditions and the FMI.

### 4.4. Validation

**Figure 18:**
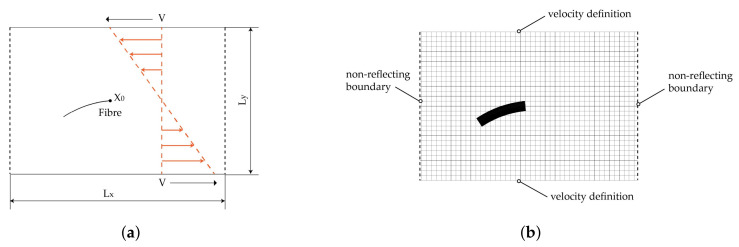
Numerical setup for the validation model. (**a**) Initial configuration. (**b**) Mesh division of the validation model.

The above results are a phenomenological comparison of different modelling methods for the deformation of fibres in shear flow. To further validate the reliability of the modelling method, the model of Takemura et al. [44] (hereinafter called Takemura’s model) was reconstructed. This model investigates the deformation of a single elastic fibre in shear flow. Figure 18a shows the initial configuration of the shear flow domain and the initial position of the fibre. The flow domain is 9 mm long ($L_{x}$) and 3 mm wide ($L_{y}$). In order to establish a shear rate of 10 s^−1^ (*G*), the top and bottom sides of the flow domain are defined with opposing flow velocities of identical magnitude *V* (15 mm/s). $X_{0}$ is the right end point of the fibre and positioned at the centre point of the fluid domain. The initial shape of the fibre is a circular arc segment of 28.65 ${}^{\circ }$, as in [44]. The length of the fibre ($L_{f}$) is 1 mm. It should be noted that Takemura’s model was two-dimensional, neglecting the thickness direction. The replication ALE model features a domain thickness of 0.3 mm. Thus, coordinates of $X_{0}$ are (4.5, 1.5, 0.15). In Takemura’s model, the fibre was discretised by 21 nodes with no volume. However, for the beam elements to be used in the ALE model a diameter needs to be defined. The diameter *D* can be converted from the Young’s modulus *E* and bending stiffness *K* of the fibre given in Takemura’s model as follows:

(7) K=E·I,

(8) $$I=\frac{\pi D^{4}}{64}\;,$$

where *I* is the second moment of area.

In agreement with Takemura’s model, the fibre is discretised into 20 beams with 21 integration nodes and the grid size of the flow domain is 0.1 mm. Figure 18b illustrates the mesh division of the validation model. In addition to the top and bottom boundaries being defined with velocities, the left and right boundaries are set with non-reflecting boundary conditions to minimise dynamic effects and the boundaries due to the high shear rates in the fluid domain. Information on mesh and material parameters is given in Table 9 and Table 10.

**Figure 19:**
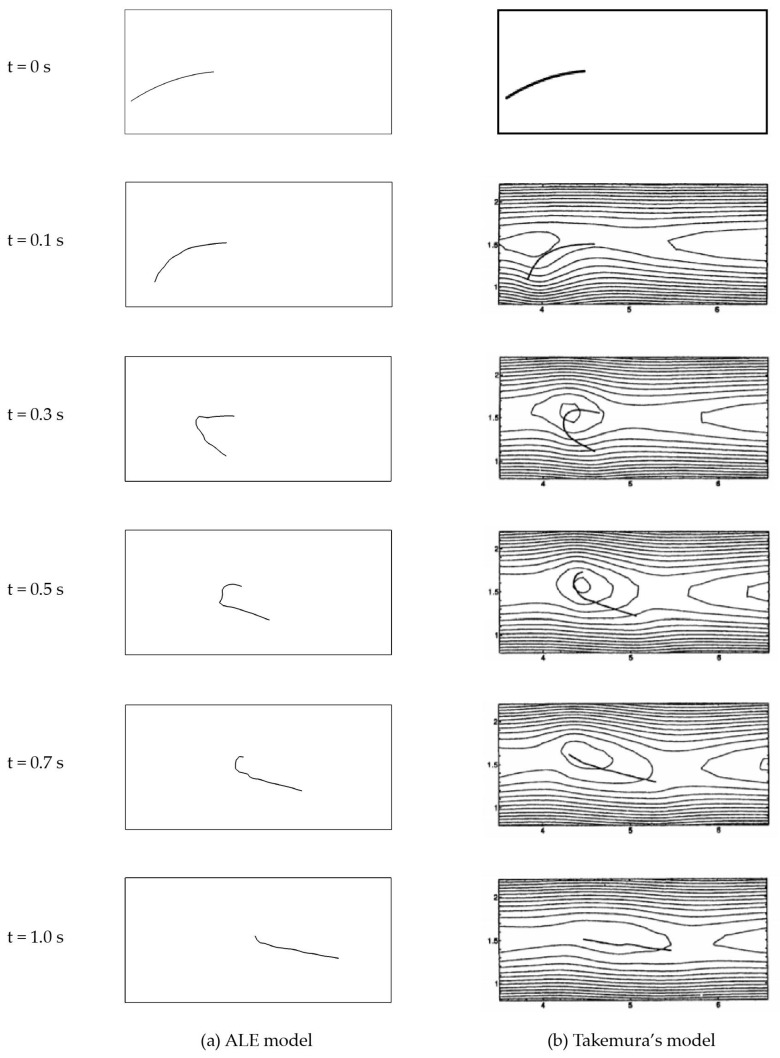
Evolutions of the fibre configuration. (**a**) ALE model. (**b**) Model in [44] (Figure 4).

The simulation results are presented in Figure 19 In general, the model shows a springy rotation in accordance to the experimental observation of [19] and summarized in [45]. In comparison to the results of Takemura’s model, the comparison shows that up to t= 0.5 s the fibre deformation is in close agreement for both result sets. After that timestamp, the fibre in the ALE model still remains partially bent, while the fibre in Takemura’s model is already nearly straight. At t= 1 s, the fibre of ALE model exhibits more rigid body displacement. This difference may be due to the quality of fibre input parameters, as the density of the fibre was not given in Takemura’s model. Furthermore, the time step may have an effect. In Takemura’s model, the time step (Δt) was set to a value of $5.0\times 10^{-6}$ s, which cannot be adjusted exactly for the ALE model. However, the close agreement of both models qualitatively validates the feasibility of the modelling method with regards to fibre deformation in shear flow.

### 4.5. Rheological FSI Model

**Figure 20:**
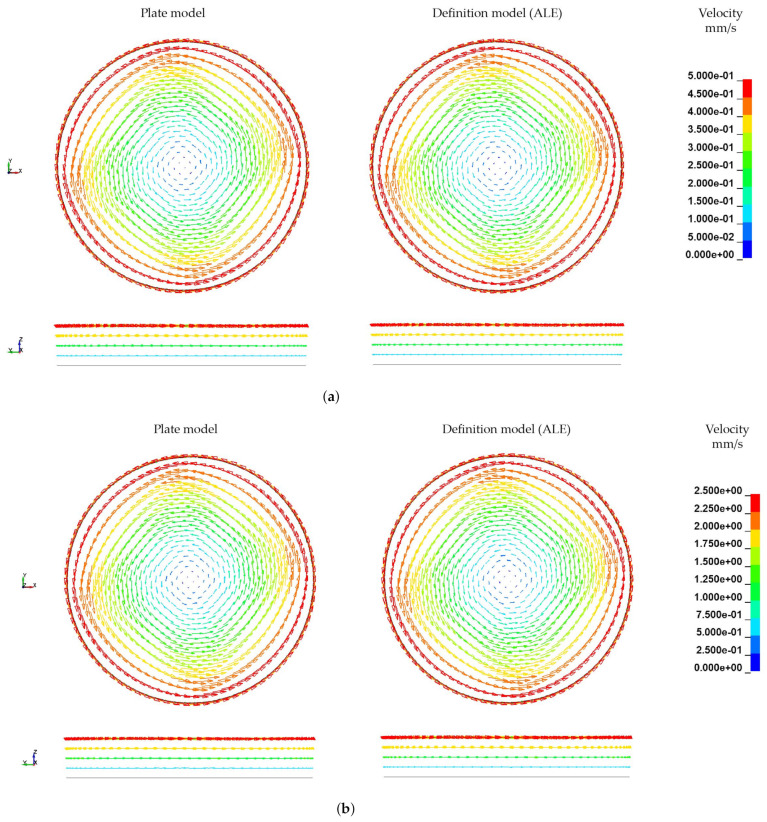

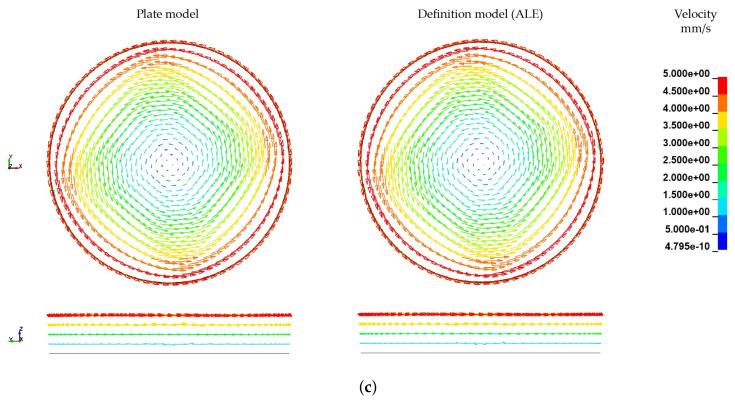
Comparison of velocity distribution at different timestamps without any scaling. (**a**) t = 0.04 s. (**b**) t = 0.2 s. (**c**) t = 0.4 s.

The simulations using the ALE approach are carried out without time or mass scaling. The angular velocity ω is increased linearly starting from zero. Resulting velocity fields of the rheological ALE models are illustrated in Figure 20. Therein the FSI induced velocity boundary conditions (left; corresponding to the plate model in Section 4.3) are compared with direct velocity boundary conditions (right) at three different timestamps. The directly defined boundary condition yields a significantly more regular velocity field over the rheological melt volume. Although the flow of the FSI boundary condition model is considerably disturbed by the plate-fluid-interaction’s influence, the global velocity field is similar to the direct boundary condition model. Both model’s velocity fields are converging over the simulation time with the FSI boundary condition model approaching the solution of the direct boundary condition model. It can be concluded, that the coupling of plate and melt in the FSI boundary condition leading to the same velocities as the calculated and directly applied ones. Furthermore, computational effort is reduced and mesh dependencies between the plate and the ALE domain leading to numerical singularities are eliminated. Furthermore, a mesh dependency of velocity fields was observed. Although for constant radii and heights constant velocities are expected at arbitrary angular coordinates, the results exhibit fan-shaped velocity distributions for both models (90° rotational symmetric). Those irregularities are caused by a variation of element size in the cylindrical melt volume. Those varying element sizes are unavoidable due to a circular cross-section has to be meshed using only elements with quadratic cross-sections.

Due to the homogenous velocity profile, further investigations are carried out with the direct velocity boundary conditions. Based on the results it was concluded that both models are equally capable of calculating resultant velocity distributions, especially in the perspective of modelling the FSI between melt and fibre. A crucial benefit resulting from those findings is the significant reduction of run times through the replacement of plate–melt interactions by direct boundary conditions. Thus, the original run time of 24 h 44 min was reduced to 7 h 21 min (running on 12 parallel CPUs).

**Figure 21:**
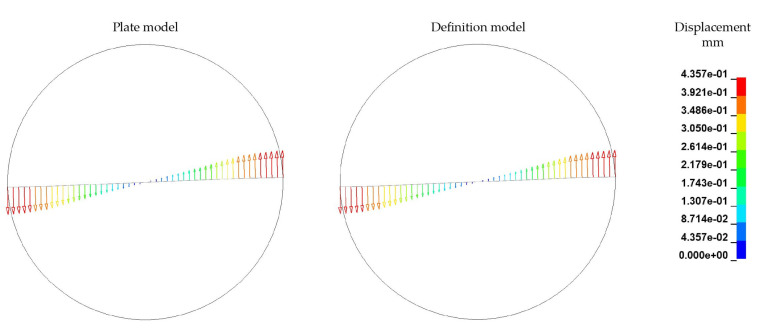
Comparison of fibre displacement and deformation with ALE approach after *T* = 0.4 s and ω= 0.4 s^−1^.

The embedded fibre itself exhibits only a slight bending deformation with a maximum at the fibre endings in the rheological FSI model (for the ALE modelling approach) while rotating (Figure 21) at ω= 0.4 s^−1^. The displacement of the rotational dominated motion of the plate model after 0.4 s is calculated to be 0.435 mm, while in the definition model the maximal displacement is 0.436 mm. For both models the deformation behaviour are similar. There are three potential influencing factors for this deviation of the numerical model to the theoretical considerations of the bending behaviour (cf. Figure 3):

- The ALE modelling approach does not take NSE into account;
- Missing boundary conditions at the circumference of the cylindrical melt volume to account for irregularities of the experimental setup;
- Lower viscous forces due to higher shear rates at the fibre endings.

The ICFD model’s major advantage is the precise modelling of structural deformations. This is achieved by the inclusion of the NSE and the implicit time integration which allows to minimise inertia effects. However, a critical disadvantage is the limited modelling capabilities in terms of structure-structure-interactions, i.e., between different fibres. Therefore, the ICFD approach has a significant limitation considering the investigated FSI problem. Therefore the ICFD approach is not considered for the rheological FSI models.

#### 4.5.1. Velocities and Boundary Conditions

**Figure 22:**
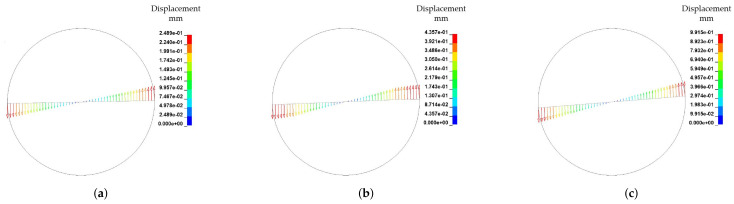
Fibre displacement by different angle velocity ω at T= 0.4 s. (**a**) Fibre displacement by ω=0.2 s^−1^. (**b**) Fibre displacement by ω=0.4 s^−1^ (Reference). (**c**) Fibre displacement by ω=0.8 s^−1^.

An evaluation of the sensitivity of the developed modelling approach was carried out by simulations with different angular velocity ramps in a linear ramp from zero to ω at T= 0.4 s. The resultant deformations are presented in Figure 22. As expected, the deformations of the fibres differ and with increasing ω the deformation increases. In contrast to the reference of ω= 0.4 s^−1^, bending areas of the fibre endings are smaller. Therefore, the rotational effects dominate for both remaining velocities. Due to the shear rate dependent viscosity, the deformation increases non linearly in contrast to angular velocities. The shear rate $\dot{\gamma }$ for the reference model at the fibre endings amounts 1.25 s^−1^. Therefore, the viscosity results in 138 Pa · s, whereas the viscosity at the rotation centre is 545.7 Pa · s. For ω= 0.8 s^−1^ the shear rate at the fibre endings amounts 2.5 s^−1^ with a viscosity of 106 Pa · s. Therefore, the different resultant deformation behaviour resulting from effects of drag force relations and viscous forces according to velocity profile in radial direction.

#### 4.5.2. Dependency of Mesh Refinement

**Figure 23:**
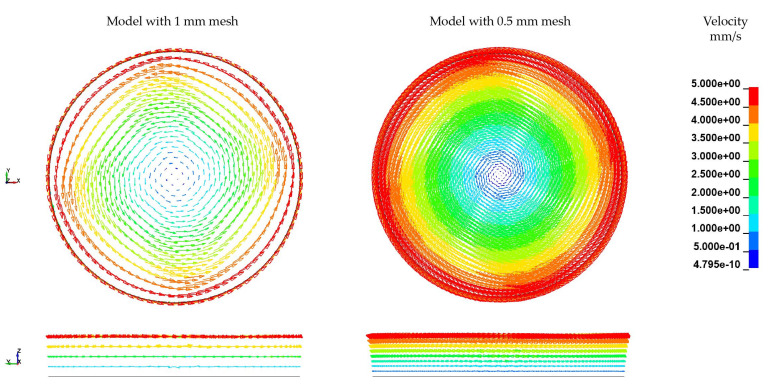
Comparison of velocity distribution by different mesh fineness.

Based on the observed velocity profile for the two FSI models, the mesh dependency of the velocity and the resultant deformation of the solid structure are investigated. In Figure 23 the mesh of the rheological FSI model is further refined from a characteristic element length $l_{e}$ of 1 mm to 0.5 mm. In Table 11, the mesh information of the refined model and the reference model is given. The boundary conditions kept constant for the refined model in accordance to the reference model.

**Figure 24:**
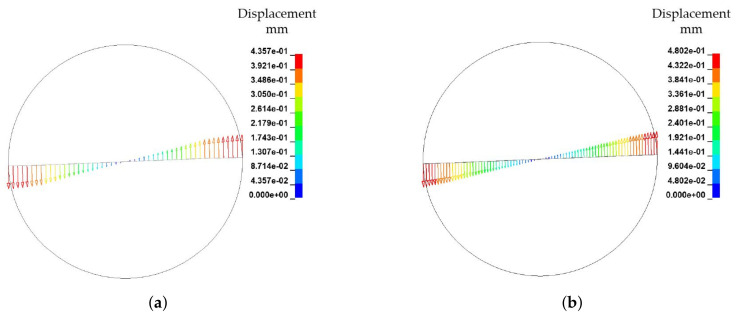
Comparison of fibre displacement and deformation by different mesh fineness. (**a**) Mesh size = 1 mm. (**b**) Mesh size = 0.5 mm.

With decreasing mesh size, the dependency of the velocity field on the mesh becomes evident leading to a more pronounced fan-shaped velocity distribution (Figure 23). Since the ALE model is based on a pre-defined finite element mesh, this dependency is difficult to avoid. However, by mesh refinement, the deviations in the velocity distribution can indeed be reduced to some extent, because more points with speed information are offered. In terms of fibre displacement and deformation, there is also a small difference in the results from different models with different mesh sizes (Figure 24). The maximum displacement of the fibres has been slightly increased by mesh refinement. In terms of overall deformation, the bending of the fibre is slightly reduced. These differences arise from the change in the velocity field due to the refinement of the fluid mesh on the one hand, and from the refinement of the mesh of the fibres itself on the other.

## 5. Discussion and Conclusions

In the present paper a modelling strategy for investigations of the FSI between high viscous melt and single fibre is developed. Therefore, simplified models addressing different investigation aspects (Table 4) are used to investigate the numerical methods of ALE and Incompressible Computational Fluid Dynamics (ICFD) in LS-Dyna. The ICFD method with NSE-based solver is used as cross-reference and for validation of the simplified ALE method. Within the models, different element types (beam, 3D solid and discrete elements) for representation of flexible fibres are investigated. The beam and discrete elements are well suited for contact modelling of solid structures (fibre–fibre interaction) in a fluid domain, whereas the 3D solid elements exhibit a significant dependency on mesh refinements. For the numerical description of FSI, two model setups with a laminar and a shear flow environment are used. In both, only the usage of beam and solid elements result in a good agreement of structural deformations and flow velocities for both methods. In contrast, discrete elements led to a unrealistic non disturbed flow profile. Therefore, discrete elements were not considered in further investigations. Tip force and deflection are in the same order of magnitude for both, beam and 3D solid elements. Nevertheless, beam elements lead to a transient response until a steady state is achieved. Due to these results, beam elements are favoured for fibre modelling purposes.

For the shear flow model, velocity induced deformations of the flexible structures lead to the expected bending behaviour of fibres (vertical model, Figure 9b) and the translations of fibres are in accordance to the velocity profile (horizontal model, Figure 9c). The vertical model was also subject of further investigations with respect to the reduction of computational effort as well as an alternative method for applying the shear velocity. The direct definition of velocity conditions at the upper side without an explicit modelling of a FSI between a rigid plate and the polymer melt led to a similar velocity profile with considerably decreased computational effort. Furthermore, a validation of the proposed modelling strategy is performed by a single fibre in a shear flow according to [44]. The fibre deformation shows similar deformation behaviour in comparison to the observation of [19,45].

Based on the findings, the resultant rheological FSI model was implemented for the ALE approach. The ALE model with a direct velocity definition exhibits a fibre rotation and slight bending at the fibre ends superimposed by the rotation. The model shows a sensitivity on the variation of rotational velocities. With respect to the proposed velocity profile and the viscosity-shear rate table, the viscous force can lead to an inhomogeneous force distribution along the fibre. Therefore, the influence of coupling and boundary effects has to be considered further. Due to the normal force in rheometer measurements, the melt is compressed and squeezed out in radial direction at the boundary areas. This leads to a changed velocity profile and has to be considered by appropriate velocity definitions.

Due to long time measurements mass and time scaling have to be considered in further work to reduce the computational effort. It can be concluded, that the scaling of shear and strain rate effects also influence the penalty-based coupling approach, which leads to different coupling forces. The scaling of viscosity and shear rate for corresponding scaling factors has to be investigated in detail. The application of this modelling strategy on other material configurations (e.g., carbon fibres) to investigate the differences in the FSI behaviour will be carried out in further work. Due to the smaller diameter of the fibres the coupling approach and the mesh quality of both fibre and matrix has to be investigated. Furthermore, anisotropic material behaviour has to be considered. Since the shear flow leads to a fibre bending, an implementation of a material model taking the bending modulus of the fibre into account seems reasonable. In further work the bending modulus will be determined by cantilever bending test setups.

In conclusion, a modelling strategy with the ALE approach for investigations of FSI was developed. This strategy allows a simulation for single fibres and fibre bundles. The results show high sensitivity of fibre deformation for boundary effects and conditions. For resource efficient modelling a velocity boundary condition is used instead of a tool–matrix interaction setup. Fibres are modelled as a beam chain. Time and mass scaling were also investigated. Resultant displacements and forces of the solid structure were evaluated. Further work, should focus on an experimental setup design for validation. Furthermore, the flow profile in rheometer tests without fibres has to be investigated in more detail. The experimentally determined velocity profile can than be transferred into the FSI model.

## Figures and Tables

**Table 1:** Glass fibre engineering constants and geometric parameters.

Constant	Scalar	Unit
Young’s modulus *E*	73	GPa
Poisson’s ratio ν	0.3	-
Diameter *D* (N=83)	14.31±1.9	μm

**Table 2:** Parameters of the material model for the thermoplastic matrix for the ALE approach.

Keyword	Parameter		
*MAT_ALE_02-ALE_VISCOUS	** ρ **	**PC**	**MULO**
	7.575 × 10${}^{-10}$	−150	DEFINE_CURVE
*EOS_GRUNEISEN	**C**	**S1**	$\gamma_{EOS}$
	1 × 10${}^{6}$	1.4	0.06718

**Table 3:** Parameters of the CROSS—model for ICFD approach.

$\eta_{0}$	$\eta_{\infty }$	Time Constant	Power Index
545.7	20.6	7.13	0.57

**Table 4:** Model evolution for the development of a modelling strategy and summarised findings.

Modelling Setup	Collision Model	Laminar Channel Flow	Shear Flow	FSI Model
Visualisation	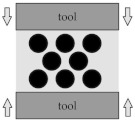	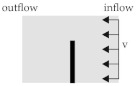	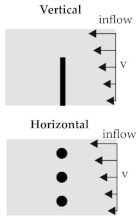	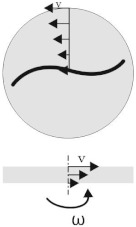
Numerical setup	Section 3.2.1	Section 3.2.2	Section 3.2.3	Section 3.3
Scope of investigation	Modelling of solids: Beam, 3D solids, DEM Contact formulation and accuracy	Comparison ALE and ICFD Velocity profile around solid Influence of solid modelling	Modelling strategy Boundary conditions Sensitivity of ALE method	Deformation of solid Influence of boundary condition Study of convergence
Results	Section 4.1	Section 4.2	Section 4.3 and Section 4.4	Section 4.5
Main findings	Mesh depended 3D solid modelling Reduction of dynamic effects using volume redundancy	DEM not suitable ALE and ICFD equally suited Beam and 3D solids equally suited	ALE favourable Phenomenologically sound interaction between fluid and beam Direct velocity definition computationally more effective Validation (Section 4.4)	Rotation dominated deformation Deformation depends on velocity

**Table 5:** Numerical parameters of the three different discretisation methods for the collision model.

Model	Fibre			Matrix			Tool		
	Type	Domain	Quantity	$l_{e}$	Domain	Quantity	$l_{e}$	Domain	Quantity
Beam	Beam	Solid	10 pro fibre	0.2 mm	ALE	35,700	0.5 mm	Solid	1200
DEM	DEM	Solid	11 pro fibre						
Solid	3D Solid	Solid	10/40/160 pro fibre						

**Table 6:** Numerical parameters for the laminar channel flow model.

Method	Model	Fibre		Matrix		
		Type	Quantity	$l_{e}$	Domain	Quantity
ALE	Beam	Beam	5	1 mm	ALE	1400
ALE	Solid	3D Solid	625		ALE	1400
ICFD	Solid	3D Solid	625		CFD	1310

**Table 7:** Numerical parameters for the shear flow model.

Fibre			Matrix			Plate		
Type	Domain	Quantity	Type	Domain	Quantity	Type	Domain	Quantity
Beam	Solid	40	0.25 mm	ALE	12,800	0.2 mm	Solid	1936

**Table 8:** Numerical setup for the rheological FSI model.

Fibre			Matrix		
Type	Domain	Quantity	$l_{e}$	Domain	Quantity
Beam	Solid	50	1 mm	ALE	3080

**Table 9:** Mesh characteristics of the validation model.

Fibre			Fluid		
Type	Domain	Quantity	$l_{e}$	Domain	Quantity
Beam	Solid	20	0.1 mm	ALE	8100

**Table 10:** Material parameter of the validation model.

	Constant	Scalar	Unit
Fibre	Young’s modulus *E*	0.001	MPa
	Density $\varrho_{F}$	2.0 × 10${}^{-9}$	ton/mm^3^
	Bending stiffness *K*	7.5 × 10${}^{-8}$	ton·mm^3^/s^2^
	Diameter *D*	0.2	mm
	Length $L_{f}$	1	mm
Fluid	Density $\varrho_{D}$	1.0 × 10${}^{-9}$	ton/mm^3^
	Viscosity μ	2.0 × 10${}^{-7}$	MPa·s

**Table 11:** Numerical setup for the refined rheological FSI model.

Model	Fibre			Matrix			Elapsed Time
	Type	Domain	Quantity	$l_{e}$	Domain	Quantity	
refined model	Beam	Solid	80	0.5 mm	ALE	26,384	69 h 8 min
reference model	Beam	Solid	50	1.0 mm	ALE	3080	7 h 22 min

## Data Availability

Not applicable.

## Abbreviations

The following abbreviations are used in this manuscript:

2D	Two dimensional
3D	Three dimensional
ALE	Arbitrary Lagrangian Eulerian
CF	carbon fibre
CFD	Computational Fluid Dynamics
CBIS	CONSTRAINT_BEAM_IN_SOLID
CLIS	CONSTRAINT_LAGRANGIAN_IN_SOLID
CPU	Central processing unit
DEM	Discrete Element Method
DMA	dynamic mechanical analysis
EOS	Equation of state
FMI	fibre-matrix-interaction
FSI	fluid-structure-interaction
GF	glass fibre
ICFD	Incompressible Computational Fluid Dynamics
LCM	liquid composite moulding
MS	multiple solver
NSE	Navier–Stokes-Equation
PMC	polymer matrix composites
PP	polypropylene
TPC	thermoplastic composites

## Appendix A. LS-Dyna Keywords for Plate Model

The appendix shows a supplemental material for the rheological FSI model with the FSI induced velocity definition (plate model) The appendix is the LS-Dyna keyword file. Every keyword is introduced with *.
